# 2-(4-Fluoro­phen­yl)-1-phenyl-1*H*-benzimidazole

**DOI:** 10.1107/S1600536812035155

**Published:** 2012-08-15

**Authors:** K. Jayamoorthy, S. Rosepriya, A. Thiruvalluvar, J. Jayabharathi, R. J. Butcher

**Affiliations:** a Department of Chemistry, Annamalai University, Annamalai Nagar 608 002, Tamilnadu, India; b PG Research Department of Physics, Rajah Serfoji Government College (Autonomous), Thanjavur 613 005, Tamilnadu, India; c Department of Chemistry, Howard University, 525 College Street NW, Washington, DC 20059, USA

## Abstract

In the title mol­ecule, C_19_H_13_FN_2_, the benzimidazole unit is close to planar [maximum deviation = 0.0342 (9) Å] and forms dihedral angles of 58.94 (3) and 51.43 (3)° with the phenyl and fluoro­benzene rings, respectively; the dihedral angle between the phenyl and fluoro­benzene rings is 60.17 (6)°. In the crystal, three C—H⋯F hydrogen bonds and two weak C—H⋯π inter­actions involving the fused benzene ring lead to a three-dimensional architecture.

## Related literature

For linear and non-linear optical properties of benzimidazole compounds, see: Cross *et al.* (1995 ▶); Bu *et al.* (1996 ▶); Dirk *et al.* (1990 ▶). For a related structure, see: Rosepriya *et al.* (2011 ▶).
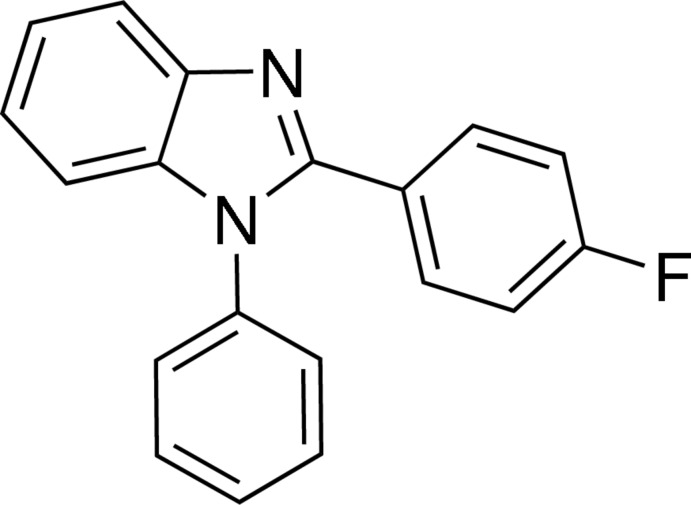


## Experimental

### 

#### Crystal data

C_19_H_13_FN_2_
*M* *_r_* = 288.31
Monoclinic, 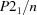

*a* = 8.7527 (4) Å
*b* = 10.1342 (4) Å
*c* = 17.0211 (6) Å
β = 104.187 (4)°
*V* = 1463.75 (11) Å^3^
*Z* = 4
Mo *K*α radiation
μ = 0.09 mm^−1^
*T* = 123 K
0.47 × 0.42 × 0.15 mm

#### Data collection

Agilent Xcalibur Ruby Gemini diffractometer
Absorption correction: multi-scan (*CrysAlis PRO*; Agilent, 2012 ▶) *T*_min_ = 0.961, *T*_max_ = 1.000
13721 measured reflections
7347 independent reflections
5352 reflections with *I* > 2σ(*I*)
*R*_int_ = 0.031

#### Refinement

*R*[*F* ^2^ > 2σ(*F* ^2^)] = 0.063
*wR*(*F* ^2^) = 0.160
*S* = 1.04
7347 reflections
199 parameters
H-atom parameters constrained
Δρ_max_ = 0.52 e Å^−3^
Δρ_min_ = −0.22 e Å^−3^

### 

Data collection: *CrysAlis PRO* (Agilent, 2012 ▶); cell refinement: *CrysAlis PRO*; data reduction: *CrysAlis PRO*; program(s) used to solve structure: *SIR2004* (Burla *et al.*, 2005 ▶); program(s) used to refine structure: *SHELXL97* (Sheldrick, 2008 ▶); molecular graphics: *ORTEP-3* (Farrugia, 1997 ▶) and *PLATON* (Spek, 2009 ▶); software used to prepare material for publication: *PLATON*.

## Supplementary Material

Crystal structure: contains datablock(s) global, I. DOI: 10.1107/S1600536812035155/tk5141sup1.cif

Structure factors: contains datablock(s) I. DOI: 10.1107/S1600536812035155/tk5141Isup2.hkl

Supplementary material file. DOI: 10.1107/S1600536812035155/tk5141Isup3.cdx

Supplementary material file. DOI: 10.1107/S1600536812035155/tk5141Isup4.cml

Additional supplementary materials:  crystallographic information; 3D view; checkCIF report

## Figures and Tables

**Table 1:** Hydrogen-bond geometry (Å, °) *Cg*2 is the centroid of the fused benzene ring (C4–C9).

*D*—H⋯*A*	*D*—H	H⋯*A*	*D*⋯*A*	*D*—H⋯*A*
C4—H4⋯F4^i^	0.93	2.46	3.3640 (14)	164
C7—H7⋯F4^ii^	0.93	2.43	3.3058 (13)	157
C26—H26⋯F4^iii^	0.93	2.52	3.4348 (14)	166
C16—H16⋯*Cg*2^iv^	0.93	2.75	3.5443 (12)	144
C22—H22⋯*Cg*2^v^	0.93	2.80	3.5245 (13)	136

Symmetry codes: (i) 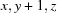; (ii) 
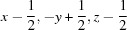
; (iii) 
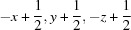
; (iv) 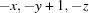; (v) 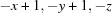.

**Fig. 1.**
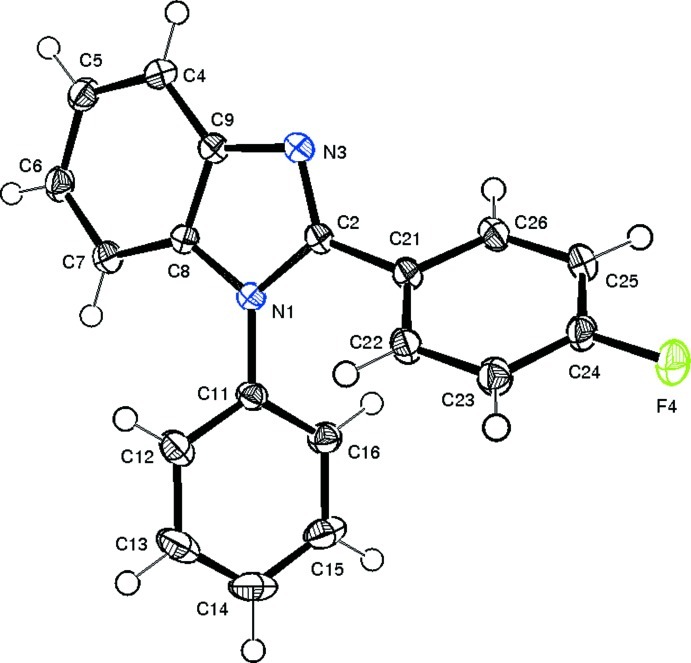
The molecular structure of the title compound, with displacement ellipsoids drawn at the 50% probability level. H atoms are shown as small spheres of arbitrary radius.

**Fig. 2.**
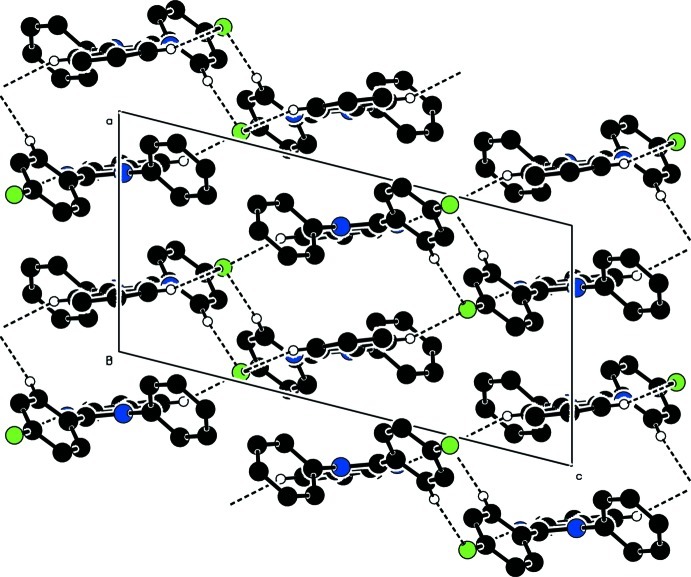
The packing of the title compound, viewed down the b axis. Dashed lines indicate hydrogen bonds. H atoms not involved in hydrogen bonding have been omitted.

## supplementary crystallographic information

### Comment

Benzimidazole based chromophores have received increasing attention due to their distinctive linear, non-linear optical properties and also due to their excellent thermal stability in guest-host systems (Cross *et al.*, 1995). The imidazole ring can be easily tailored to accommodate functional groups, which allows the covalent incorporation of the NLO chromophores into polyamides leading to NLO side chain polymers (Bu *et al.*, 1996). Most π-conjugated systems play a major role in determining second-order NLO response (Dirk *et al.*, 1990). Since our group is doing research in organic light emitting devices (OLEDs), we are interested in using the title compound as a ligand in the preparation of Ir(III) complexes and in studying the photophysical properties of these complexes. Rosepriya *et al.* (2011) have reported a related crystal structure, namely 1-(4-Methylbenzyl)-2-(4-methylphenyl)-1*H*-benzimidazole.

In the title molecule, C_19_H_13_FN_2_ (Fig. 1), the benzimidazole unit is almost planar [maximum deviation = 0.0342 (9) Å for C6]. The dihedral angles between the planes of the benzimidazole and the phenyl and the fluorobenzene groups are 58.94 (3) and 51.43 (3)°, respectively. The dihedral angle between the planes of the phenyl and the fluorobenzene rings is 60.17 (6)°. Intermolecular C4—H4···F4, C7—H7···F4 and C26—H26···F4 hydrogen bonds and weak C16—H16···π and C22—H22···π interactions involving the fused benzene ring are found in the crystal structure (Fig. 2, Table 1).

### Experimental

To *N*-phenyl-*o*-phenylenediamine (3.128 g, 17 mmol) in ethanol (10 ml) was added 4-fluorobenzaldehyde (1.9 ml, 17 mmol) and ammonium acetate (3 g) over about 1 h while maintaining the temperature at 353 K. The reaction mixture was refluxed until the completion of reaction, as monitored by TLC. The reaction mixture was extracted with dichloromethane. The solid that separated out was purified by column chromatography using petroleum ether: ethyl acetate as the eluent. Yield: 2.47 g (50%). The title compound was dissolved in acetonitrile and allowed to slowly evaporate for two days to obtain crystals suitable for X-ray diffraction studies.

### Refinement

The H atoms were positioned geometrically and allowed to ride on their parent atoms, with C—H = 0.93 Å and *U*_iso_(H) = 1.2*U*_eq_(C).

### Crystal data

**Table:**

C_19_H_13_FN_2_	*F*(000) = 600
*M**_r_* = 288.31	*D*_x_ = 1.308 Mg m^−^^3^
Monoclinic, *P*2_1_/*n*	Melting point: 369 K
Hall symbol: -P 2yn	Mo *K*α radiation, λ = 0.71073 Å
*a* = 8.7527 (4) Å	Cell parameters from 3202 reflections
*b* = 10.1342 (4) Å	θ = 3.1–37.6°
*c* = 17.0211 (6) Å	µ = 0.09 mm^−^^1^
β = 104.187 (4)°	*T* = 123 K
*V* = 1463.75 (11) Å^3^	Plate, colourless
*Z* = 4	0.47 × 0.42 × 0.15 mm

### Data collection

**Table:**

Agilent Xcalibur Ruby Gemini diffractometer	7347 independent reflections
Radiation source: Enhance (Mo) X-ray Source	5352 reflections with *I* > 2σ(*I*)
Graphite monochromator	*R*_int_ = 0.031
Detector resolution: 10.5081 pixels mm^-1^	θ_max_ = 37.7°, θ_min_ = 3.1°
ω scans	*h* = −12→15
Absorption correction: multi-scan (*CrysAlis PRO*; Agilent, 2012)	*k* = −12→17
*T*_min_ = 0.961, *T*_max_ = 1.000	*l* = −28→24
13721 measured reflections	

### Refinement

**Table:**

Refinement on *F*^2^	Primary atom site location: structure-invariant direct methods
Least-squares matrix: full	Secondary atom site location: difference Fourier map
*R*[*F*^2^ > 2σ(*F*^2^)] = 0.063	Hydrogen site location: inferred from neighbouring sites
*wR*(*F*^2^) = 0.160	H-atom parameters constrained
*S* = 1.04	*w* = 1/[σ^2^(*F*_o_^2^) + (0.0685*P*)^2^ + 0.2726*P*] where *P* = (*F*_o_^2^ + 2*F*_c_^2^)/3
7347 reflections	(Δ/σ)_max_ = 0.001
199 parameters	Δρ_max_ = 0.52 e Å^−^^3^
0 restraints	Δρ_min_ = −0.22 e Å^−^^3^

### Special details

**Table:**

Geometry. Bond distances, angles *etc*. have been calculated using the rounded fractional coordinates. All su's are estimated from the variances of the (full) variance-covariance matrix. The cell e.s.d.'s are taken into account in the estimation of distances, angles and torsion angles
Refinement. Refinement of *F*^2^ against ALL reflections. The weighted *R*-factor *wR* and goodness of fit *S* are based on *F*^2^, conventional *R*-factors *R* are based on *F*, with *F* set to zero for negative *F*^2^. The threshold expression of *F*^2^ > 2σ(*F*^2^) is used only for calculating *R*-factors(gt) *etc*. and is not relevant to the choice of reflections for refinement. *R*-factors based on *F*^2^ are statistically about twice as large as those based on *F*, and *R*- factors based on ALL data will be even larger.

### Fractional atomic coordinates and isotropic or equivalent isotropic displacement parameters (Å^2^)

**Table:**

	*x*	*y*	*z*	*U*_iso_*/*U*_eq_	
F4	0.45822 (10)	−0.05502 (7)	0.23042 (4)	0.0296 (2)	
N1	0.25329 (11)	0.44506 (9)	−0.01000 (5)	0.0181 (2)	
N3	0.32447 (11)	0.53932 (9)	0.11392 (5)	0.0204 (2)	
C2	0.30699 (12)	0.42744 (10)	0.07314 (6)	0.0181 (2)	
C4	0.27719 (14)	0.77382 (11)	0.06432 (7)	0.0224 (3)	
C5	0.22041 (14)	0.84918 (11)	−0.00490 (7)	0.0241 (3)	
C6	0.16727 (14)	0.79006 (11)	−0.08140 (7)	0.0242 (3)	
C7	0.17379 (14)	0.65470 (11)	−0.09192 (6)	0.0227 (3)	
C8	0.23331 (12)	0.57999 (10)	−0.02222 (6)	0.0184 (2)	
C9	0.28005 (12)	0.63684 (10)	0.05527 (6)	0.0192 (2)	
C11	0.21724 (12)	0.34608 (10)	−0.07164 (6)	0.0181 (2)	
C12	0.29236 (14)	0.34944 (12)	−0.13497 (6)	0.0250 (3)	
C13	0.25650 (17)	0.25270 (14)	−0.19472 (7)	0.0326 (4)	
C14	0.14865 (18)	0.15428 (13)	−0.19126 (7)	0.0342 (4)	
C15	0.07463 (16)	0.15197 (12)	−0.12781 (8)	0.0296 (3)	
C16	0.10831 (13)	0.24826 (11)	−0.06771 (6)	0.0222 (3)	
C21	0.34251 (12)	0.29683 (10)	0.11128 (6)	0.0184 (2)	
C22	0.43985 (13)	0.20730 (11)	0.08453 (6)	0.0207 (3)	
C23	0.47933 (13)	0.08832 (11)	0.12460 (6)	0.0226 (3)	
C24	0.41834 (13)	0.06137 (11)	0.19050 (6)	0.0216 (3)	
C25	0.31950 (14)	0.14570 (11)	0.21819 (6)	0.0240 (3)	
C26	0.28287 (14)	0.26534 (11)	0.17816 (6)	0.0224 (3)	
H4	0.31207	0.81317	0.11491	0.0269*	
H5	0.21753	0.94056	−0.00049	0.0289*	
H6	0.12661	0.84318	−0.12621	0.0290*	
H7	0.14030	0.61573	−0.14271	0.0273*	
H12	0.36522	0.41522	−0.13727	0.0299*	
H13	0.30549	0.25419	−0.23741	0.0391*	
H14	0.12581	0.08983	−0.23133	0.0410*	
H15	0.00225	0.08581	−0.12547	0.0355*	
H16	0.05837	0.24707	−0.02538	0.0266*	
H22	0.47838	0.22748	0.03966	0.0248*	
H23	0.54500	0.02834	0.10762	0.0271*	
H25	0.27886	0.12332	0.26204	0.0288*	
H26	0.21808	0.32514	0.19596	0.0269*	

### Atomic displacement parameters (Å^2^)

**Table:**

	*U*^11^	*U*^22^	*U*^33^	*U*^12^	*U*^13^	*U*^23^
F4	0.0397 (4)	0.0227 (3)	0.0241 (3)	0.0008 (3)	0.0033 (3)	0.0082 (3)
N1	0.0235 (4)	0.0161 (3)	0.0140 (3)	−0.0019 (3)	0.0035 (3)	−0.0001 (3)
N3	0.0259 (4)	0.0195 (4)	0.0158 (3)	−0.0011 (3)	0.0051 (3)	−0.0005 (3)
C2	0.0208 (4)	0.0193 (4)	0.0144 (3)	−0.0009 (3)	0.0047 (3)	0.0010 (3)
C4	0.0266 (5)	0.0188 (4)	0.0221 (4)	−0.0028 (4)	0.0065 (4)	−0.0029 (4)
C5	0.0275 (5)	0.0183 (4)	0.0280 (5)	−0.0008 (4)	0.0096 (4)	0.0009 (4)
C6	0.0279 (5)	0.0209 (5)	0.0235 (4)	0.0007 (4)	0.0059 (4)	0.0055 (4)
C7	0.0279 (5)	0.0217 (5)	0.0174 (4)	−0.0007 (4)	0.0032 (3)	0.0028 (3)
C8	0.0214 (4)	0.0170 (4)	0.0167 (4)	−0.0021 (3)	0.0045 (3)	0.0002 (3)
C9	0.0214 (4)	0.0197 (4)	0.0167 (4)	−0.0021 (3)	0.0048 (3)	−0.0003 (3)
C11	0.0203 (4)	0.0186 (4)	0.0148 (3)	0.0007 (3)	0.0030 (3)	−0.0010 (3)
C12	0.0285 (5)	0.0289 (5)	0.0192 (4)	0.0028 (4)	0.0091 (4)	0.0010 (4)
C13	0.0437 (7)	0.0362 (7)	0.0192 (4)	0.0128 (6)	0.0102 (5)	−0.0029 (4)
C14	0.0464 (8)	0.0263 (6)	0.0244 (5)	0.0114 (5)	−0.0017 (5)	−0.0089 (4)
C15	0.0329 (6)	0.0203 (5)	0.0307 (5)	0.0001 (4)	−0.0017 (4)	−0.0051 (4)
C16	0.0236 (5)	0.0207 (4)	0.0211 (4)	−0.0011 (4)	0.0034 (3)	−0.0015 (4)
C21	0.0211 (4)	0.0194 (4)	0.0144 (3)	−0.0013 (3)	0.0039 (3)	0.0009 (3)
C22	0.0230 (4)	0.0222 (5)	0.0180 (4)	0.0003 (4)	0.0073 (3)	0.0027 (3)
C23	0.0244 (5)	0.0219 (5)	0.0218 (4)	0.0019 (4)	0.0062 (4)	0.0024 (4)
C24	0.0266 (5)	0.0189 (4)	0.0170 (4)	−0.0022 (4)	0.0010 (3)	0.0042 (3)
C25	0.0322 (5)	0.0244 (5)	0.0167 (4)	−0.0023 (4)	0.0086 (4)	0.0027 (4)
C26	0.0278 (5)	0.0233 (5)	0.0178 (4)	0.0009 (4)	0.0090 (4)	0.0008 (3)

### Geometric parameters (Å, º)

**Table:**

F4—C24	1.3633 (13)	C21—C22	1.3952 (15)
N1—C2	1.3890 (13)	C21—C26	1.4005 (15)
N1—C8	1.3877 (14)	C22—C23	1.3867 (15)
N1—C11	1.4298 (13)	C23—C24	1.3825 (15)
N3—C2	1.3186 (13)	C24—C25	1.3787 (16)
N3—C9	1.3908 (13)	C25—C26	1.3893 (15)
C2—C21	1.4735 (14)	C4—H4	0.9300
C4—C5	1.3897 (16)	C5—H5	0.9300
C4—C9	1.3976 (15)	C6—H6	0.9300
C5—C6	1.4050 (16)	C7—H7	0.9300
C6—C7	1.3864 (16)	C12—H12	0.9300
C7—C8	1.3961 (14)	C13—H13	0.9300
C8—C9	1.4052 (14)	C14—H14	0.9300
C11—C12	1.3939 (15)	C15—H15	0.9300
C11—C16	1.3883 (15)	C16—H16	0.9300
C12—C13	1.3919 (17)	C22—H22	0.9300
C13—C14	1.384 (2)	C23—H23	0.9300
C14—C15	1.3891 (19)	C25—H25	0.9300
C15—C16	1.3920 (17)	C26—H26	0.9300
			
C2—N1—C8	106.15 (8)	F4—C24—C25	118.13 (9)
C2—N1—C11	128.03 (9)	C23—C24—C25	123.67 (10)
C8—N1—C11	125.75 (8)	C24—C25—C26	117.52 (10)
C2—N3—C9	104.91 (8)	C21—C26—C25	120.69 (10)
N1—C2—N3	113.10 (9)	C5—C4—H4	121.00
N1—C2—C21	123.13 (9)	C9—C4—H4	121.00
N3—C2—C21	123.76 (9)	C4—C5—H5	119.00
C5—C4—C9	117.69 (10)	C6—C5—H5	119.00
C4—C5—C6	121.32 (10)	C5—C6—H6	119.00
C5—C6—C7	121.80 (10)	C7—C6—H6	119.00
C6—C7—C8	116.45 (10)	C6—C7—H7	122.00
N1—C8—C7	131.97 (9)	C8—C7—H7	122.00
N1—C8—C9	105.45 (8)	C11—C12—H12	121.00
C7—C8—C9	122.51 (9)	C13—C12—H12	121.00
N3—C9—C4	129.50 (9)	C12—C13—H13	120.00
N3—C9—C8	110.38 (9)	C14—C13—H13	120.00
C4—C9—C8	120.12 (9)	C13—C14—H14	120.00
N1—C11—C12	119.29 (10)	C15—C14—H14	120.00
N1—C11—C16	119.75 (9)	C14—C15—H15	120.00
C12—C11—C16	120.96 (10)	C16—C15—H15	120.00
C11—C12—C13	118.90 (11)	C11—C16—H16	120.00
C12—C13—C14	120.69 (12)	C15—C16—H16	120.00
C13—C14—C15	119.87 (12)	C21—C22—H22	120.00
C14—C15—C16	120.29 (12)	C23—C22—H22	120.00
C11—C16—C15	119.30 (10)	C22—C23—H23	121.00
C2—C21—C22	121.34 (9)	C24—C23—H23	121.00
C2—C21—C26	118.84 (9)	C24—C25—H25	121.00
C22—C21—C26	119.75 (10)	C26—C25—H25	121.00
C21—C22—C23	120.23 (10)	C21—C26—H26	120.00
C22—C23—C24	118.14 (10)	C25—C26—H26	120.00
F4—C24—C23	118.21 (10)		
			
C8—N1—C2—N3	−0.88 (13)	C6—C7—C8—N1	178.40 (12)
C8—N1—C2—C21	179.88 (10)	C6—C7—C8—C9	1.94 (17)
C11—N1—C2—N3	−178.02 (10)	N1—C8—C9—N3	−1.25 (12)
C11—N1—C2—C21	2.74 (17)	N1—C8—C9—C4	178.72 (10)
C2—N1—C8—C7	−175.66 (12)	C7—C8—C9—N3	176.02 (10)
C2—N1—C8—C9	1.24 (12)	C7—C8—C9—C4	−4.00 (17)
C11—N1—C8—C7	1.56 (19)	N1—C11—C12—C13	−179.97 (12)
C11—N1—C8—C9	178.46 (10)	C16—C11—C12—C13	0.00 (17)
C2—N1—C11—C12	−123.95 (12)	N1—C11—C16—C15	−179.68 (10)
C2—N1—C11—C16	56.07 (16)	C12—C11—C16—C15	0.33 (17)
C8—N1—C11—C12	59.44 (15)	C11—C12—C13—C14	−0.32 (19)
C8—N1—C11—C16	−120.54 (12)	C12—C13—C14—C15	0.3 (2)
C9—N3—C2—N1	0.11 (13)	C13—C14—C15—C16	0.1 (2)
C9—N3—C2—C21	179.34 (10)	C14—C15—C16—C11	−0.36 (18)
C2—N3—C9—C4	−179.25 (12)	C2—C21—C22—C23	176.01 (10)
C2—N3—C9—C8	0.72 (12)	C26—C21—C22—C23	−0.90 (16)
N1—C2—C21—C22	50.81 (15)	C2—C21—C26—C25	−177.04 (10)
N1—C2—C21—C26	−132.26 (11)	C22—C21—C26—C25	−0.06 (17)
N3—C2—C21—C22	−128.35 (12)	C21—C22—C23—C24	0.67 (16)
N3—C2—C21—C26	48.58 (16)	C22—C23—C24—F4	−179.40 (10)
C9—C4—C5—C6	0.15 (18)	C22—C23—C24—C25	0.53 (17)
C5—C4—C9—N3	−177.21 (11)	F4—C24—C25—C26	178.48 (10)
C5—C4—C9—C8	2.83 (17)	C23—C24—C25—C26	−1.45 (17)
C4—C5—C6—C7	−2.2 (2)	C24—C25—C26—C21	1.18 (17)
C5—C6—C7—C8	1.13 (18)		

### Hydrogen-bond geometry (Å, º)

Cg2 is the centroid of the fused benzene ring (C4–C9).

**Table:**

*D*—H···*A*	*D*—H	H···*A*	*D*···*A*	*D*—H···*A*
C4—H4···F4^i^	0.93	2.46	3.3640 (14)	164
C7—H7···F4^ii^	0.93	2.43	3.3058 (13)	157
C26—H26···F4^iii^	0.93	2.52	3.4348 (14)	166
C16—H16···*Cg*2^iv^	0.93	2.75	3.5443 (12)	144
C22—H22···*Cg*2^v^	0.93	2.80	3.5245 (13)	136

Symmetry codes: (i) *x*, *y*+1, *z*; (ii) *x*−1/2, −*y*+1/2, *z*−1/2; (iii) −*x*+1/2, *y*+1/2, −*z*+1/2; (iv) −*x*, −*y*+1, −*z*; (v) −*x*+1, −*y*+1, −*z*.
